# Supplementary material for: One-Carbon Metabolism in Alzheimer’s Disease and Parkinson’s Disease Brain Tissue
Source: Nutrients. 2022 Jan 29;14(3):599. doi: 10.3390/nu14030599 (PMC8838558; doi:10.3390/nu14030599)
Supplement: Supplementary file 1 [file nutrients-14-00599-s001.zip › nutrients-1564958 - Supplementary Material B.pdf]

Supplementary Materials

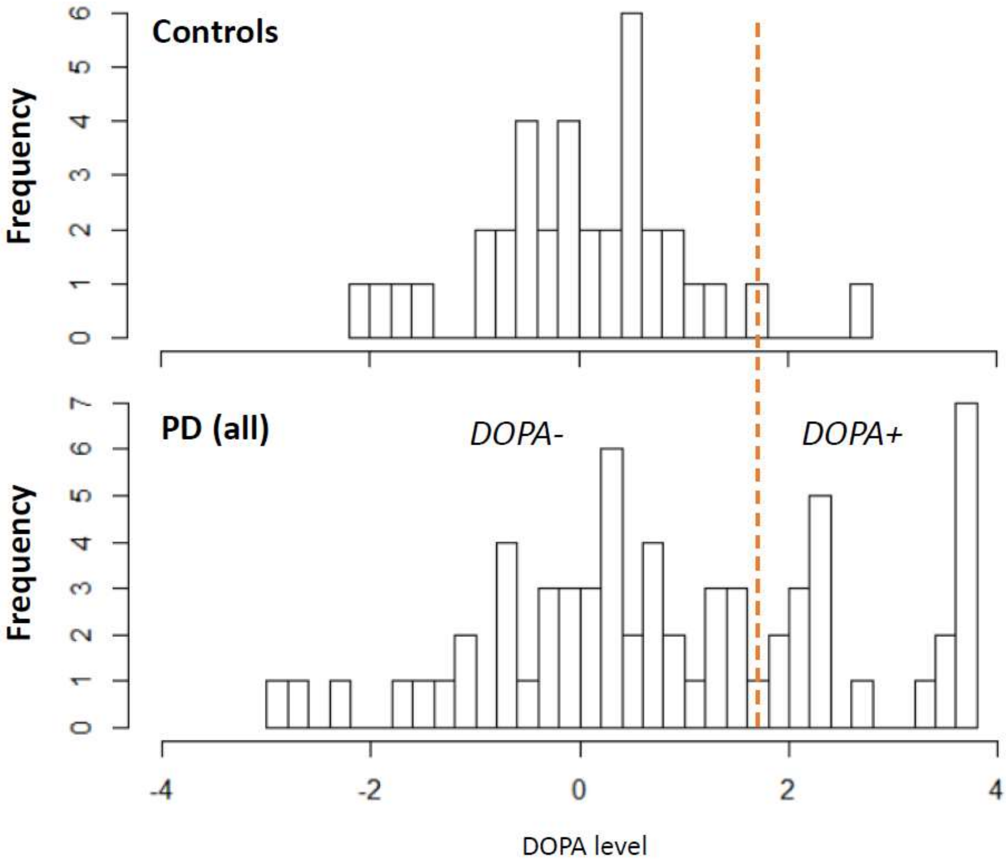

Figure S1. Histograms of normalized DOPA concentrations among controls and PD subjects. The dashed orange line shows the threshold selected for detection of PD subjects with levodopa medication active in the system at the time of death.
